# Supplementary material for: Multi‐dataset identification of innovative feature genes and molecular mechanisms in keratoconus
Source: J Cell Mol Med. 2024 Sep 19;28(18):e70079. doi: 10.1111/jcmm.70079 (PMC11412914; doi:10.1111/jcmm.70079)
Supplement: Supplementary file 2 — Material S2. [file JCMM-28-e70079-s002.zip › 7.GSEA analysis/5.RNF217-AS1/KEGG_RIBOSOME.html]

Details for gene set KEGG\_RIBOSOME[GSEA]

|  || Dataset | RNF217-AS1.group.cls#h\_versus\_l |
| Phenotype | group.cls#h\_versus\_l |
| Upregulated in class | h |
| GeneSet | KEGG\_RIBOSOME |
| Enrichment Score (ES) | 0.28639472 |
| Normalized Enrichment Score (NES) | 1.0639477 |
| Nominal p-value | 0.34468937 |
| FDR q-value | 1.0 |
| FWER p-Value | 0.998 |
Table: GSEA Results Summary

  

Fig 1: Enrichment plot: KEGG\_RIBOSOME      
 Profile of the Running ES Score & Positions of GeneSet Members on the Rank Ordered List

  

| SYMBOL | TITLE | RANK IN GENE LIST | RANK METRIC SCORE | RUNNING ES | CORE ENRICHMENT || 1 | RPS24 | na | 6833 | 0.158 | -0.1241 | Yes |
| 2 | RPL32 | na | 9615 | 0.093 | -0.1612 | Yes |
| 3 | RPS27 | na | 11272 | 0.066 | -0.1783 | Yes |
| 4 | RPS6 | na | 11627 | 0.061 | -0.1604 | Yes |
| 5 | RPL28 | na | 11631 | 0.061 | -0.1323 | Yes |
| 6 | RPS21 | na | 12132 | 0.054 | -0.1219 | Yes |
| 7 | RPL22L1 | na | 13257 | 0.039 | -0.1363 | Yes |
| 8 | RPL3L | na | 14788 | 0.021 | -0.1705 | Yes |
| 9 | RPS9 | na | 14927 | 0.020 | -0.1653 | Yes |
| 10 | RSL24D1 | na | 15666 | 0.012 | -0.1809 | Yes |
| 11 | RPL36A | na | 16130 | 0.007 | -0.1908 | Yes |
| 12 | RPL36AL | na | 16153 | 0.007 | -0.1881 | Yes |
| 13 | RPL39 | na | 16479 | 0.004 | -0.1957 | Yes |
| 14 | RPL26 | na | 16512 | 0.003 | -0.1950 | Yes |
| 15 | RPL23 | na | 16752 | 0.001 | -0.2014 | Yes |
| 16 | RPL24 | na | 16770 | 0.001 | -0.2014 | Yes |
| 17 | RPS4X | na | 16818 | 0.000 | -0.2026 | Yes |
| 18 | RPL38 | na | 16923 | -0.001 | -0.2053 | Yes |
| 19 | FAU | na | 17096 | -0.003 | -0.2090 | Yes |
| 20 | RPS29 | na | 17108 | -0.003 | -0.2081 | Yes |
| 21 | RPL36 | na | 17140 | -0.003 | -0.2076 | Yes |
| 22 | RPS15A | na | 17182 | -0.003 | -0.2072 | Yes |
| 23 | RPL12 | na | 17219 | -0.004 | -0.2066 | Yes |
| 24 | RPS12 | na | 17255 | -0.004 | -0.2056 | Yes |
| 25 | RPS3 | na | 17334 | -0.005 | -0.2056 | Yes |
| 26 | RPL8 | na | 17553 | -0.007 | -0.2087 | Yes |
| 27 | RPLP2 | na | 17563 | -0.007 | -0.2057 | Yes |
| 28 | RPS25 | na | 17813 | -0.009 | -0.2087 | Yes |
| 29 | RPL4 | na | 17825 | -0.009 | -0.2048 | Yes |
| 30 | RPL31 | na | 17916 | -0.010 | -0.2027 | Yes |
| 31 | RPL34 | na | 17926 | -0.010 | -0.1983 | Yes |
| 32 | RPL14 | na | 17957 | -0.010 | -0.1944 | Yes |
| 33 | RPL11 | na | 18162 | -0.012 | -0.1946 | Yes |
| 34 | RPS28 | na | 18253 | -0.013 | -0.1911 | Yes |
| 35 | RPS15 | na | 18259 | -0.013 | -0.1852 | Yes |
| 36 | RPL21 | na | 18303 | -0.014 | -0.1801 | Yes |
| 37 | RPL19 | na | 18333 | -0.014 | -0.1746 | Yes |
| 38 | RPS8 | na | 18442 | -0.015 | -0.1708 | Yes |
| 39 | RPS3A | na | 18455 | -0.015 | -0.1641 | Yes |
| 40 | RPL35 | na | 18475 | -0.015 | -0.1576 | Yes |
| 41 | RPS18 | na | 18495 | -0.015 | -0.1510 | Yes |
| 42 | RPS7 | na | 18648 | -0.017 | -0.1475 | Yes |
| 43 | RPL18 | na | 18665 | -0.017 | -0.1401 | Yes |
| 44 | RPS13 | na | 18706 | -0.017 | -0.1332 | Yes |
| 45 | RPL35A | na | 18774 | -0.018 | -0.1267 | Yes |
| 46 | RPL17 | na | 18826 | -0.019 | -0.1196 | Yes |
| 47 | RPL5 | na | 18837 | -0.019 | -0.1112 | Yes |
| 48 | RPL41 | na | 18849 | -0.019 | -0.1029 | Yes |
| 49 | RPL27 | na | 18940 | -0.020 | -0.0964 | Yes |
| 50 | RPL13A | na | 18977 | -0.020 | -0.0883 | Yes |
| 51 | RPL9 | na | 19010 | -0.020 | -0.0799 | Yes |
| 52 | RPS16 | na | 19094 | -0.021 | -0.0726 | Yes |
| 53 | RPL23A | na | 19307 | -0.023 | -0.0679 | Yes |
| 54 | RPLP1 | na | 19337 | -0.024 | -0.0578 | Yes |
| 55 | RPL13 | na | 19357 | -0.024 | -0.0473 | Yes |
| 56 | RPL7 | na | 19390 | -0.024 | -0.0371 | Yes |
| 57 | RPL7A | na | 19464 | -0.025 | -0.0276 | Yes |
| 58 | RPS27A | na | 19478 | -0.025 | -0.0163 | Yes |
| 59 | RPS17 | na | 19483 | -0.025 | -0.0048 | Yes |
| 60 | RPS2 | na | 19581 | -0.026 | 0.0045 | Yes |
| 61 | RPS11 | na | 19586 | -0.026 | 0.0164 | Yes |
| 62 | RPL15 | na | 19602 | -0.026 | 0.0282 | Yes |
| 63 | RPL10A | na | 19620 | -0.026 | 0.0399 | Yes |
| 64 | RPL30 | na | 19622 | -0.026 | 0.0521 | Yes |
| 65 | RPS26 | na | 19626 | -0.026 | 0.0643 | Yes |
| 66 | RPL26L1 | na | 19632 | -0.027 | 0.0765 | Yes |
| 67 | RPL10L | na | 19709 | -0.027 | 0.0869 | Yes |
| 68 | RPS10 | na | 19741 | -0.028 | 0.0987 | Yes |
| 69 | MRPL13 | na | 19751 | -0.028 | 0.1113 | Yes |
| 70 | RPL37A | na | 19766 | -0.028 | 0.1237 | Yes |
| 71 | UBA52 | na | 19783 | -0.028 | 0.1362 | Yes |
| 72 | RPL18A | na | 19880 | -0.029 | 0.1467 | Yes |
| 73 | RPL6 | na | 19886 | -0.029 | 0.1599 | Yes |
| 74 | RPL29 | na | 19891 | -0.029 | 0.1732 | Yes |
| 75 | RPS5 | na | 20172 | -0.032 | 0.1798 | Yes |
| 76 | RPS23 | na | 20427 | -0.034 | 0.1883 | Yes |
| 77 | RPS4Y1 | na | 20493 | -0.035 | 0.2026 | Yes |
| 78 | RPS20 | na | 20503 | -0.035 | 0.2185 | Yes |
| 79 | RPL22 | na | 20662 | -0.037 | 0.2309 | Yes |
| 80 | RPL3 | na | 20668 | -0.037 | 0.2477 | Yes |
| 81 | RPLP0 | na | 20909 | -0.039 | 0.2589 | Yes |
| 82 | RPS19 | na | 21167 | -0.042 | 0.2709 | Yes |
| 83 | RPL37 | na | 21326 | -0.043 | 0.2864 | Yes |
| 84 | RPS27L | na | 24641 | -0.085 | 0.2301 | No |
| 85 | RPL27A | na | 27288 | -0.132 | 0.2148 | No |
Table: GSEA details [plain text format]

  

Fig 2: KEGG\_RIBOSOME      
 Blue-Pink O' Gram in the Space of the Analyzed GeneSet

  

Fig 3: KEGG\_RIBOSOME: Random ES distribution      
 Gene set null distribution of ES for **KEGG\_RIBOSOME**
